# Thidiazuron: New Trends and Future Perspectives to Fight *Xylella fastidiosa* in Olive Trees

**DOI:** 10.3390/antibiotics11070947

**Published:** 2022-07-14

**Authors:** Alessia Catalano, Jessica Ceramella, Domenico Iacopetta, Annaluisa Mariconda, Elisabetta Scali, Maria Grazia Bonomo, Carmela Saturnino, Pasquale Longo, Stefano Aquaro, Maria Stefania Sinicropi

**Affiliations:** 1 Department of Pharmacy-Drug Sciences, University of Bari Aldo Moro, 70126 Bari, Italy; 2 Department of Pharmacy, Health and Nutritional Sciences, University of Calabria, 87036 Cosenza, Italy; jessica.ceramella@unical.it (J.C.); domenico.iacopetta@unical.it (D.I.); stefano.aquaro@unical.it (S.A.); s.sinicropi@unical.it (M.S.S.); 3 Department of Science, University of Basilicata, 85100 Potenza, Italy; annaluisa.mariconda@unibas.it (A.M.); mariagrazia.bonomo@unibas.it (M.G.B.); carmela.saturnino@unibas.it (C.S.); 4 Department of Health Sciences, Magna Graecia University, 88100 Catanzaro, Italy; elisabettascali@libero.it; 5 Department of Chemistry and Biology, University of Salerno, Via Giovanni Paolo II, 132, 84084 Fisciano, Italy; plongo@unisa.it

**Keywords:** antimicrobials, diarylurea, bis-arylurea, cytokinin, *Xylella fastidiosa*, lavender, rosemary, olives, almonds, Italy, Apulia

## Abstract

These days, most of our attention has been focused on the COVID-19 pandemic, and we have often neglected what is happening in the environment. For instance, the bacterium *Xylella fastidiosa* re-emerged as a plant pathogen of global importance in 2013 when it was first associated with an olive tree disease epidemic in Italy, called Olive Quick Decline Syndrome (OQDS), specifically caused by *X. fastidiosa* subspecies *pauca* ST53, which affects the Salento olive trees (Apulia, South-East Italy). This bacterium, transmitted by the insect *Philaenus spumarius,* is negatively reshaping the Salento landscape and has had a very high impact in the production of olives, leading to an increase of olive oil prices, thus new studies to curb this bacterium are urgently needed. Thidiazuron (TDZ), a diphenylurea (*N*-phenyl-1,2,3-thiadiazol-5-yl urea), has gained considerable attention in recent decades due to its efficient role in plant cell and tissue culture, being the most suitable growth regulator for rapid and effective plant production in vitro. Its biological activity against bacteria, fungi and biofilms has also been described, and the use of this low-cost compound to fight OQDS may be an intriguing idea.

## 1. Introduction

**Figure 1:**
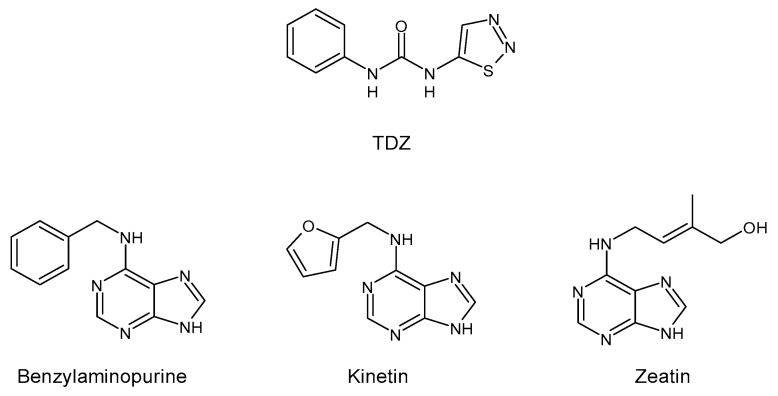
Structure of TDZ and other cytokinins.

Thidiazuron (*N*-phenyl-*N′*-1,2,3-thiadiazol-5-ylurea, TDZ) is among the most active cytokinin-like substances used for woody plant tissue culture [1]. It belongs to the plant growth regulators (PGRs) and was registered in 1976 by Schering AG (Berlin, Germany) as a cotton defoliant, with the name SN 49537 [2]. In 1985, studies in cotton (*Gossypium hirsutum* L. cv Stoneville 519) seedlings suggested that TDZ-induced leaf abscission may be mediated, at least in part, by an increase in endogenous ethylene evolution [3]. The chemical structure of TDZ is shown in Figure 1. Unlike the other adenine-type cytokinins such as benzylaminopurine, kinetin, or zeatin, TDZ does not contain the purine ring. It belongs to diarylureas or bis-arylureas, an interesting class of compounds with various biological activities [4,5].

In 1982, Mok et al., showed the high promoting growth activity of cytokinin-dependent callus cultures of *Phaseolus lunatus* cv. Kingston exerted by TDZ [6]. TDZ demonstrated cytokinin activity higher than that of zeatin. Then, Thomas and Katterman [7] demonstrated that TDZ was able to stimulate maximum soybean callus growth and radish cotyledon expansion, as well as tobacco plantlet regeneration. Numerous studies have shown that TDZ affects endogenous cytokinin and auxin production, and therefore morphogenetic recognition of cells and tissues by down-regulating various genes related with auxin regulation and transport, as well as cytokinin response [8]. In comparison to other PGRs, TDZ has been defined as the most effective and efficient on enhancing the levels of important metabolites in many medicinal plants [9,10] and not only in organogenesis in different plants [11,12]. DZ was used for the cyclic shoot multiplication of tulip, as it enables the production of virus-free stock plants and speeds up breeding [13]; it was also applied to the in vitro propagation of saffron, where it induced somatic embryogenesis [14], and it promoted in vitro shoot regeneration from callus of barley (*Hordeum vulgare* L.) and wheat (*Triticum aestivum* L.) [15,16]. As a potent synthetic growth regulator, TDZ presents a wide array of in vitro and in vivo applications in plants, including prevention of leaf yellowing, enhanced photosynthetic activity, breaking of bud dormancy, fruit ripening, as well as proliferation of adventitious shoots, callus production, and induction of somatic embryogenesis [17]. Moreover, among the various biologically activities exerted by diarylureas [18,19], TDZ specifically demonstrated antitumor and antimicrobial activities [20,21], and was also suggested as a potential agent for diagnosis of solid tumors, such as cervix cancer [22].

**Figure 2:**
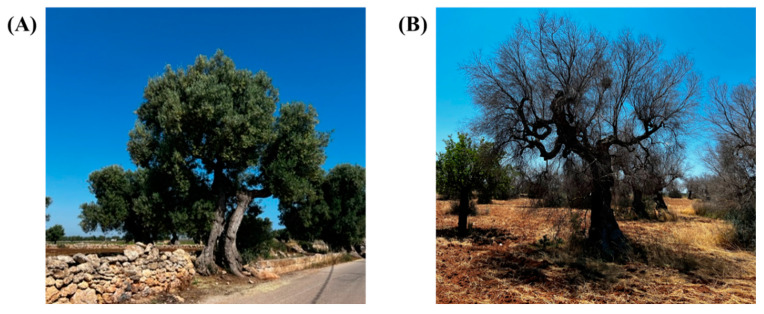
Olive trees undamaged (**A**) and severely damaged (**B**) by *Xfp* in the Apulia region.

*Xylella fastidiosa* (*Xf*) is a non-spore-forming Gram-negative phytopathogenic bacterium belonging to the *Xanthomonadaceae* family, colonizing the xylem vessels of almost 600 plant species—275 genera and 85 families [23]. Though the world is concentrated on coronavirus disease 2019 (COVID-19) [24], the current impact of *Xf* in many parts of the world is now considerable [25] and causes the disease in a number of important crops and plants within natural ecosystems. The *Xf* symptoms are leaf scorching, wilting of the foliage, defoliation, chlorosis or bronzing along the leaf margin, and dwarfing [26]. Most of the infected species do not demonstrate remarkable alterations, but destructive diseases occur in important crops such as olives [27], grapevine [28], citrus [29], and stone fruits [30,31], as well as in numerous ornamental and forest species [32]. Most notable among these diseases are Pierce’s disease (PD) of grapevine, Citrus Variegated Chlorosis (CVC), Almond Leaf Scorch Disease (ALSD), Oleander Leaf Scorch (OLS) and Olive Quick Decline Syndrome (OQDS) [33]. *Xf* has been found in more than 500 plant species, especially the perennial ones [34,35]. A dramatic outbreak of *Xf* subsp. *pauca* (*Xfp*) strain ST53, namely CoDiRO (Complesso del Disseccamento Rapido dell’Olivo, meaning OQDS), decimating olive trees was discovered in 2013 in Apulia, Southern Italy [36]. The meadow spittlebug *Philaenus spumarius* L. (1978) (Hemiptera: Auchenorrhyncha: Aphrophoridae) is considered the main vector in the *Xf* outbreak [37]. To date, this disease is limited to the Salento region. Figure 2 shows olive trees undamaged (A) and severely damaged (B) by *Xfp*. The areas of Barletta-Andria-Trani BAT (Apulia) and Potenza PZ (Basilicata, Apulia bordering region) and the north of Salento are pathogen-free.

Several studies are being carried out to understand the underlying causes of *Xf* emergence and spread among olive trees. Nevertheless, several important questions regarding *Xf* remain unsolved, e.g., how it interacts with the different host plants, with the plant immune system and how it influences the host’s microbiome [38]. Sanitation of infected olive trees is unfeasible [39], and very few phyto-therapeutics were evaluated to mitigate OQDS disease. Among the disease control attempts, the usage of commercially available Dentamet^®^, a Zn/Cu citric acid biocomplex, has been assessed as a foliar treatment able to reduce the *Xfp* cell concentration in olive trees [40]. New metabolomics approaches have been proposed for the diagnosis of OQDS markers in olive tree leaves [41,42]; however, OQDS remains a major concern, and new studies are needed to stop the diffusion of this bacterium.

The diffusion and transmission of *Xf* to olive trees is due to insects, and *P. spumarius* L. is now considered the major epidemiologically relevant vector currently responsible for *Xf* spread in Europe [43]. Researchers from Italy, France and the USA have attributed the arrival in Italy of *Xf* bacterium, in 2008, to a coffee plant probably coming from Costa Rica, and then adapted to infect the olive trees in Apulia [44]. The spread of *Xf* is rapid, difficult to halt, and seems to be directed to other regions in the south of Italy, including Calabria [45]. In this review, we summarize the problems related to *Xf* outbreak, particularly in Italy, considering the bacterium itself as the vector responsible for its transmission and suggesting the potential use of TDZ, which is endowed with antimicrobial and cytokinin-like activity, as well.

## 2. Thidiazuron (TDZ)

TDZ (*N*-phenyl-*N*′-1,2,3-thiadiazol-5-ylurea) is a substituted diphenylurea compound [46]. Belonging to the diarylurea class, it may be easily synthesized [47,48], and is also characterized by a low-cost [49]. It was first reported to have cytokinin activity in 1982 [50] and since then, TDZ has been used as a growth regulator for abscission of green-turgid leaves of cotton to facilitate the picking of bolls [2]. TDZ exhibits both auxin- and cytokinin-like effects on growth and differentiation of cultured explants, although, under a chemical point of view, it is totally different from commonly used auxins and cytokinins [51]. In some species, TDZ activity was about 30 times higher than zeatin activity [52] and stimulates the induction of shoot regeneration and somatic embryogenesis in some plant species [15,53]. Recently, it was studied as a growth regulator on direct shoot regeneration and production of bioactive volatile organic compounds in *Ajuga bracteosa* [54]. Moreover, an interesting activity was found in the micropropagation of *Linum usitatissimum*, commonly known as “flax” or “linseed”, an important medicinal plant that produces biologically potent lignans, used in the treatment of several human diseases. The supplementation with TDZ in the culture media efficiently activated the antioxidant system in the in vitro raised shoots, leading to maximum production of total phenolic content, total flavonoid content, antioxidant enzymes and lignans [55]. In a recent study, TDZ was shown to be effective in inducing in vitro clonal propagation of *Lagerstroemia speciosa* (L.) Pers., commonly known as ‘Pride of India’ or ‘Banaba’, belonging to the family Lythraceae, and is an important avenue tropical deciduous tree widely distributed in the Philippines, Malaysia, India, Vietnam, and China [56]. Furthermore, TDZ has induced efficient in vitro organogenesis and regeneration of *Scutellaria bornmuelleri*, a medicinal plant belonging to the *Scutellaria* genus of the Lamiaceae family, endemic in the East Azerbaijan province of Iran, used in traditional medicines to treat constipation, wound healing, and stress [57], and has increased shoot induction and proliferation rate of *Tecoma stans* L. (Bignoniaceae), commonly known as Ginger-Thomas, a plant endowed with antitumor, antioxidant, antimicrobial, antidiabetic and free radical scavenging properties [58]. Moreover, the application of TDZ inhibits the leaf yellowing in different plants, such as the one occurring after pinching potted rose plants [59]. TDZ has recently shown to enhance secondary metabolites production [60]. The most interesting activity of TDZ, which could be useful for the treatment of *Xf*, is the antibacterial one. Kumari et al. (2016) [61] studied the *Cotyledon orbiculata L.* (Crassulaceae), a succulent medicinal plant popularly known as pig’s ear, the leaf of which is used in traditional medicine to treat, soften or remove hard corns, warts and boils, and for the treatment of inflammation, toothache, earache, abscesses, skin eruptions, epilepsy and syphilis. They found that, after the treatment with TDZ, all the in vitro and ex vitro plant tissues exhibited bioactivity against both Gram-positive and Gram-negative bacteria, including *Klebsiella pneumoniae,* whereas garden-grown mother plants failed in bioactivity. A more recent study by the same group on *Eucomis autumnalis* and *Drimia robusta* showed that bulbs of *D. robusta* ex vitro-derived from solid culture with 10 µM picloram, 1 µM TDZ and 20 µM glutamine exhibited good antibacterial activity against *Enterococcus faecalis*, *Micrococcus luteus* and *Staphylococcus aureus* when compared with other treatments [62]. In a study on *Coleonema pulchellum* Williams (Rutaceae), an evergreen, erect and dense shrub, which occurs from the western to the eastern cape in South Africa, TDZ at low concentration (4.5 μM) determined the formation of a high number of normal shoots, whereas at higher concentrations (13.6 μM), showed antibacterial activity against *E. faecalis* (MIC = 1.56 mg/mL) [63], a Gram-positive organism responsible for serious infections [64]. A recent study demonstrated the antibiofilm activity of TDZ against *C. albicans*, a common human fungal pathogen that colonizes mucosa and develops biofilm in the oral cavity causing oral candidiasis. This activity was exerted by the interaction between TDZ and amino acid residues of cytochrome P450 mono-oxygenase (CYP51), acting as a new CYP51 inhibitor. TDZ treatment down-regulated the expression of genes involved in ergosterol synthesis, cell adhesion and hyphae development in *C. albicans* [21]. Despite more than 40 years of use, universal application in the environment and hundreds of scientific studies demonstrating TDZ-induced plant morphogenesis, the precise mechanism of action remains unknown. Recently, using a metabolomics approach, several hypotheses for the mechanism of action of TDZ were suggested for understanding its regulatory role in plant morphogenesis [65].

## 3. *Xylella fastidiosa* (*Xf*)

*Xf* Wells is a xylem-limited Gram-negative bacterium native to the Americas, which belongs to the family *Xanthomonadaceae* (Gammaproteobacteria); it is an obligatory colonizer of plant and insect hosts [66] and is able to form biofilms, the mechanism of which is currently under study [67]. The first to report on a disease caused by *Xf* was Newton Pierce in 1892 [68], whose studies were addressed to PD, an epidemic of vine disease in Southern California that had devastating consequences in the grape industry. More than a century later, PD remains a significant problem for the grape industry in California [69]. Then, *Xf* remained poorly characterized until the late 1970s, when it was first cultured in vitro [70]. In 1987, it emerged in Brazil and was associated with a citrus disease [71]. At the same time, another PD epidemic in Southern California devastated the local wine industry after the establishment of an invasive vector, *Homalodisca vitripennis* (Hemiptera: Cicadellidae) [72,73]. These two epidemics encouraged in-depth studies on *Xf*. In 2010, olive trees on the west coast of Salento peninsula, Italy, began to decline and die with a condition of unknown etiology that was called “OQDS” [74]. Before 2013, there were only sporadic reports of *Xf* detection in Europe [75] and its presence, particularly in Italy, was firstly described in 2013 [76]. Several molecular and pathogenic traits distinguish this bacterium from many common phytopathogenic bacteria of this family [77]. *Xf* has been well documented for its worldwide spread and infection of a broad range of plant species [78]. Specifically, OQDS is caused by *Xfp* strain ST53 and is spread by xylem-feeding insects (i.e., responsible for local spread), and through infected plant propagating materials (i.e., mainly responsible for the long-distance spread) [79]. Six different subspecies of *Xf* have been proposed [80], and 87 different sequence types have been described worldwide [81]. *Xf* was first confined to the Americas; however, international movements of infected plants for landscape planting or commercial purposes contributed to the spread and establishment of this bacterium in Europe during the last decade [82]. Other subspecies, including ST6 and ST7, have been detected in Corsica and the Provence-Alpes-Côte d’Azur and in a region of the South of France, Occitanie (Aude) and ST88 and ST89 in the PACA region [83]. Thus, preventive measures have been adopted in Europe, including inspections and diagnostic tests on imported consignments of plants and in nurseries, and this bacterium was classified as “harmful quarantine pathogen”, and more recently as one of the European priority pests (Regulation EU 2019/1702) [84]. Nevertheless, these measures failed to effectively protect the European territories, due to the biological complexity of this pathogen. Consequently, the pathogen is currently threatening olives, almonds and several other species in several outbreaks discovered mainly in southern Europe countries. The bacterium was detected in 2013 in southern Italy olives, then in 2015 was detected on ornamentals and on several Mediterranean shrubs in natural habitats in Corsica and southern mainland France; in 2016, the bacterium was detected in the Balearic Islands and mainland Spain; in late 2018, two outbreaks were found, respectively, in central Italy (Tuscany) and in Portugal. The latest reports are from France in the Occitanie region, where an outbreak was detected on lavender plants in 2020 and from Portugal on rosemary plants in 2021. Currently, mandatory checks on plant propagating materials are enforced in Europe (EU regulation 2020/1201) for the most susceptible species found in the European outbreaks, as well as on the numerous “specified plants” propagated in nurseries located in the infected, containment and buffer zones [77]. The major injury has been inflicted on the olive orchards of southern Apulia (Italy), where millions of trees died for a severe disease associated with the *Xfp* strain “De Donno”. The dramatic changes in the Mediterranean landscape and the continuously evolving situation led to the implementation of European and national (Italian and Spanish) measures to reduce the spread of the pathogen and the associated OQDS [79].

## 4. *Philaenus spumarius* L.

Sharpshooters (Hemiptera: Cicadellidae: Cicadellinae) and spittlebugs (Hemiptera: Aphrophoridae: Aphrophorinae) are vectors with a worldwide distribution and are often associated with many crops [85]. *P. spumarius* L. (1978) (Hemiptera: Auchenorrhyncha: Aphrophoridae) is considered the major epidemiologically relevant vector of *Xfp* strain ST53, responsible for the outbreak of the OQDS in Southern Italy [86,87]. Although any xylem-sap feeding insect could theoretically transmit *Xf* bacterium, only three species (Hemiptera, Aphrophoridae) have been proven to be capable of acquiring the CoDiRO strain from infected olive plants and spreading it to other plants as *P. spumarius*, *P. italosignus*, and *Neophilaenus campestris* [88], even though *Xfp* ST53 has also been found in other species of Hemiptera [89,90,91]. *P. spumarius* and other Auchenorrhyncha are known to communicate via vibrations, and indeed the possible occurrence of semiochemical communication is an interesting study recently carried out by some researchers [92]. Several differences in males and females have been found, amongst them the females of *P. spumarius* can walk significantly more at a significantly higher velocity than males. Moreover, the olfactory response of *P. spumarius* adults to two Volatile Organic Compounds (VOCs) (cis-3-hexenyl acetate and cis-3-hexen-1-ol) present in almond, olive and vine leaves were studied. VOCs were tested at different concentrations (5, 10, 20 and 30 µg/µL), and at the lowest concentration (5 µg/µL), females of *P*. *spumarius* were significantly attracted by the two VOCs, whereas at the highest concentrations (30 µg/µL), no significant differences were detected among treatments [93]. Interestingly, in another study, playbacks obtained from prerecorded *P. spumarius’* signals were shown to significantly disrupt species mating and could integrate with other techniques aimed at reducing the spread of *Xf* [94]. Finally, the importance of the climate is related not only to the bacterium itself, but also to the vector. Indeed, areas predicted as highly suitable just for the bacterium but not optimal for this vector are apparently still free of severe *Xf* outbreaks, suggesting that climate tolerances of *P. spumarius* might partly explain the current spatial pattern of *Xf* outbreaks in Europe and should always be considered in further risk assessments [95].

## 5. Differences in Olive Varieties

The ‘Ogliarola salentina’ and ‘Cellina di Nardö’ varieties are particularly sensitive to *Xf* infection and show severe symptoms [96], whereas ‘Arbosana’, ‘Arbequina’, ‘Menara’, ‘Koroneiki’ and ‘Haouzia’ may tolerate the infection by *Xf* to varying degrees. Thus far, the cultivars ‘Leccino’ and ‘FS17’ (also referred to as ‘Favolosa’) were shown to display resistance to *Xf* [97]. Intermediate resistance was reported for ‘Frantoio’, ‘Toscanina’, ‘Termite di Bitetto’, ‘Maiatica’, ‘Dolce di Cassano’, ‘Oliastro’, ‘Nociara’, and ‘Nocellara Etnea’ [98]. Resistance/tolerance to *Xfp* in the ‘Lecciana’ variety is currently under evaluation [99]. Several studies suggest the dependence of sensibility of different species to the mineral content, indeed in the low sensitive species, Mn, Cu, and Zn content is higher and Ca and Na levels are lower [100]. Moreover, the higher content of Zn and Cu both in soil and leaves found in the olive trees in northern areas of Apulia (Barletta-Andria-Trani, namely BAT province) and Basilicata, an Apulia bordering region (Potenza, PZ province), in comparison to the southern areas of Salento (LE, BR, TA provinces) could partly explain the absence of OQDS in those areas. A higher zinc content in leaves characterizes treated- versus untreated-trees [101]. Future efforts are aimed at the selection of cultivars displaying resistance to *Xf* [102].

## 6. Agrochemicals and Minerals Used for the Treatment of *Xylella fastidiosa*

Different control measures are used for the treatment of *Xf* and are summarized in the article published by EFSA [103]. What is clear is that minerals, such as zinc and copper, are useful for the treatment of this bacterium. Dentamet^®^, a biocomplex containing zinc (4%), copper (2%), and citric acid, has been used for the treatment of *Xf*-infected trees, and the earliest descriptions of its application via foliar spray have shown a reduction of *Xf*-associated disease severity; however, the time range of the application and the number of observations are limited, thus no conclusive evidence of complete eradication of the pathogen was obtained [104]. A further mid-term assessment revealed that the bacterial concentration tended to decrease in trees regularly sprayed with the biocomplex over 3–4 years [105]. A series of studies conducted in vitro [106,107,108] showed that alterations in mineral homeostasis, mainly involving zinc, copper, and calcium ions, may have significant effects on *Xf* Temecula1, responsible for PD in grapevine.

Besides the administration of zinc and copper, other strategies to control *Xf* in olive plants and employing mineral solutions have been attempted in Italy. The use of ammonium chloride sprays on OQDS-affected trees showed clear symptom reductions, but no substantial differences in the bacterial populations were observed [109]. A better well-studied control strategy for *Xf* employs *N*-acetylcysteine (NAC), a mucolytic cysteine analogue mainly used to treat human diseases [110]. It showed promising inhibitory effects on *Xf* strain 9a5c and its associated disease in sweet orange plants [111]. The treatment with NAC, especially using NAC endotherapy, in OQDS in Apulia seems to decrease disease progression, but a significant reduction in the bacterial population size has not been detected by qPCR [112]. Cattò et al. [113] studied the effect of NAC on *Xf* strain “De Donno” and found that sub-lethal concentrations of NAC had a significant effect on *Xf* biofilm formation, inducing a hyper-attaching phenotype, with potential impacts on strain virulence and vector acquisition. Recently, metal nanooxides have also been studied as carriers for the direct release of phytodrugs targeting *Xf* in olive plants. Transmission electron microscopy observations showed an alteration of the bacterial cell wall after the use of nanocarriers with calcium carbonate, which were absorbed by the olive roots and successfully translocated to conductive tissues [114]. Other authors demonstrated the antibacterial activity of NuovOlivo^®^, a natural detergent made from plants oils and extracts of multi botanical species plus sodium and calcium hydroxide, and sulfur, activated with sodium bicarbonate, improving OQDS control in both ‘Cellina di Nardò’ and ‘Ogliarola salentina’ olive groves [115].

Antimicrobial peptides (AMPs) were also suggested as alternatives to traditional compounds, because of their activity against a wide range of plant pathogens and low cytotoxicity [116]. Gomesin, a potent AMP from a tarantula spider, modulates the *Xf* gene expression profile in susceptible hosts, such as citrus trees, upon 60 min of treatment with a sublethal concentration. Moreover, the treatment of *Xf* with a sublethal concentration of gomesin before inoculation in tobacco plants correlates with a reduction in foliar symptoms, an effect probably due to the trapping of bacterial cells to fewer xylem vessels, given the enhancement in biofilm production [117]. A paratransgenic strategy that halts pathogen *Xf* transmission, using the Glassy-Winged Sharpshooter *Homalodisca vitripennis* has recently been described [118].

Moreover, several phenolic compounds, including coumarins, stilbenes and flavonoids, have been studied for their potential use against PD-associated *Xf* strains (Table 1). These compounds were effective in inhibiting *Xf* growth, showing low minimum inhibitory concentrations [119]. The study of plant-derived phenolics compounds, such as 4-methylcathecol, cathecol, veratric acid, caffeic acid, and oleuropein demonstrated the inhibitory activities against *Xf* strain “De Donno” isolated from olive plants, although it was limited to reversible bacteriostatic effects [120]. In another study, other phenolic compounds, such as gallic acid, epicatechin, and resveratrol, determined no or very low inhibition of the growth of *Xf*; however, epicatechin and gallic acid reduced cell surface adhesion. In addition, cell–cell aggregation decreased with resveratrol treatment [121].

Finally, plasma activated water (PAW) showed interesting antimicrobial potential to inactivate *Xf* cells. Only 15 min of treatment seemed to be sufficient to destroy the strain “De Donno” of *Xfp* haplotype ST53 cells in in vitro experiments [122]. However, so far, such mineral solutions and other compounds described did not lead to efficient *Xf* disease control and new products are still needed, such as antimicrobials that should overcome the phenomenon of antimicrobial resistance [123].

## 7. Summary, Outlook and Challenges

*Xfp* ST53 is an invasive Gram-negative bacterium belonging to the *Xanthomonadaceae* family, responsible for the outbreak of the OQDS, a disease causing a massive dieback of olive trees in Apulia, Southern Italy. The global distribution of this pathogen continues to increase due to anthropogenic movements of goods and plant materials. Environmental issues, such as restoration of the damaged landscape, are of crucial importance for land use development plans at regional, national and international levels. However, curbing OQDS is still a utopia. In the face of such an aggressive pathogen, it is necessary to detect and constantly monitor the most representative vectors for each area in order to promptly intervene and avoid further propagation in uncontaminated territories. Monitoring and information exchange are essential to build a levee against an uncontrolled spread of the infection. Several studies have been carried out on the *Xfp* bacterium, as well as on *P. spumarius* L., the major epidemiologically relevant vector currently responsible for OQDS spread in Italy, and on the importance of the climate for the diffusion of this disease. However, we are far from being free of this disease, and new treatments or strategies are needed. In this scenario, the study of new antibacterials may be envisaged, and our idea is the use of TDZ, a low-cost plant growth regulator that also prevents leaf yellowing, enhances photosynthetic activity, fruit ripening, as well as stimulates the proliferation of adventitious shoots, callus production, and induces somatic embryogenesis. Its interesting antimicrobial action, along with all these activities, might suggest the use of this compound for a potential treatment of OQDS.

## Figures and Tables

**Table 1:** Structure of compounds described in the text.

Structure	Name
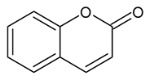	Coumarins (general structure)
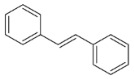	Stilbenes (general structure)
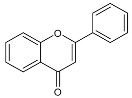	Flavonoids (general structure)
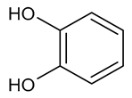	Catecol
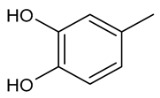	4-Methylcatecol
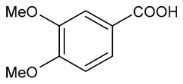	Veratric acid
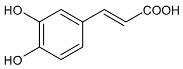	Caffeic acid
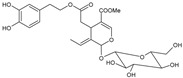	Oleuropein
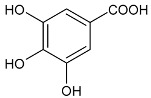	Gallic acid
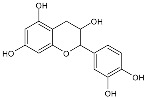	Epicatechin
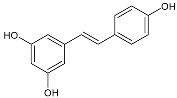	Resveratrol

## Data Availability

Not applicable.

## Abbreviations

ALSD	Almond Leaf Scorch Disease
AMPs	Antimicrobial peptides
CoDiRO	Complesso del Disseccamento Rapido dell’Olivo
COVID-19	Coronavirus disease 2019
CVC	Citrus Variegated Chlorosis
MIC	Minimal Inhibitory Concentration
OLS	Oleander Leaf Scorch
OQDS	Olive Quick Decline Syndrome
PD	Pierce’s Disease
PGRs	plant growth regulators
TDZ	Thidiazuron
VOCs	Volatile Organic Compounds
*Xf*	*Xylella fastidiosa*
*Xpf*	*Xylella fastidiosa* subspecies *pauca*

## References

[1] Lu C.-Y. (1993). The use of thidiazuron in tissue culture. Vitr. Cell. Dev. Biol. Plant.

[2] Amdt F., Rusch R., Stilfried H.V. (1976). SN 49537, a new cotton defoliant. Plant Physiol..

[3] Suttle J.C. (1985). Involvement of ethylene in the action of the cotton defoliant thidiazuron. Plant Physiol..

[4] Catalano A., Iacopetta D., Sinicropi M.S., Franchini C. (2021). Diarylureas as antitumor agents. Appl. Sci..

[5] Iacopetta D., Catalano A., Ceramella J., Saturnino C., Salvagno L., Ielo I., Drommi D., Scali E., Plutino M.R., Rosace G. (2021). The different facets of triclocarban: A review. Molecules.

[6] Mok M.C., Mok D.W.S., Armstrong D.J., Shudo K., Isogai Y., Okamoto T. (1982). Cytokinin activity of *N*-phenyl-*N’*-1,2,3-thiadiazol-5-ylurea (thidiazuron). Phytochemistry.

[7] Thomas J.C., Katterman F.R. (1986). Cytokinin activity induced by thidiazuron. Plant Physiol..

[8] Guo B., Abbasi B.H., Zeb A., Xu L.L., Wei Y.H. (2011). Thidiazuron: A multi-dimensional plant growth regulator. Afr. J. Biotechnol..

[9] Kumari A., Baskaran P., Plačková L., Omámiková H., Nisler J., Doležal K., Van Staden J. (2018). Plant growth regulator interactions in physiological processes for controlling plant regeneration and in vitro development of *Tulbaghia simmleri*. J. Plant Physiol..

[10] Kumari A., Baskaran P., van Staden J. (2017). In vitro propagation via organogenesis and embryogenesis of *Cyrtanthus mackenii*: A valuable threatened medicinal plant. Plant Cell Tissue Organ Cult..

[11] Liu X.N., Zhang X.Q., Sun J.S. (2007). Effects of cytokinins and elicitors on the production of hypericins and hyperforin metabolites in *Hypericum sampsonii* and *Hypericum perforatum*. Plant Growth Regul..

[12] Wannakrairoj S., Tefera W. (2012). Thidiazuron and other plant bioregulators for Axenic culture of siam Cardamom (Amomum krervanh Pierre ex Gangnep). Kasetsart J..

[13] Podwyszyńska M., Sochacki D., Jain S.M., Ochatt S.J. (2010). Micropropagation of Tulip: Production of Virus-Free Stock Plants. Protocols for In Vitro Propagation of Ornamental Plants, Methods in Molecular Biology.

[14] Sheibani M., Nemati S.H., Davarinejad G.H., Azghandi A.V., Habashi A.A. (2007). Induction of somatic embryogenesis in saffron using thidiazuron (TDZ). Acta Hortic..

[15] Shan X., Li D., Qu R. (2000). Thidiazuron promotes in vitro regeneration of wheat and barley. Vitr. Cell. Develo. Biol. Plant.

[16] Basile G., De Maio A.C., Catalano A., Ceramella J., Iacopetta D., Bonofiglio D., Saturnino C., Sinicropi M.S. Ancient Wheats as Promising Nutraceuticals for Human Health. Curr. Med. Chem..

[17] Dinani E.T., Shukla M.R., Turi C.E., Sullivan J.A., Saxena P.K. (2018). Thidiazuron: Modulator of morphogenesis in vitro. Thidiazuron: From Urea Derivative to Plant Growth Regulator.

[18] Catalano A., Iacopetta D., Pellegrino M., Aquaro S., Franchini C., Sinicropi M.S. (2021). Diarylureas: Repositioning from antitumor to antimicrobials or multi-target agents against new pandemics. Antibiotics.

[19] Catalano A. (2022). Diarylurea: A privileged scaffold in drug discovery and therapeutic development. Curr. Med. Chem..

[20] Enkhtaivan G., Kim D.H., Pandurangan M. (2017). Cytotoxic effect of TDZ on human cervical cancer cells. J. Photochem. Photobiol. B Biol..

[21] Harikrishnan P., Arayambath B., Jayaraman V.K., Ekambaram K., Ahmed E.A., Senthilkumar P., Ibrahim H.-I.M., Sundaresan A., Thirugnanasambantham K. (2022). Thidiazuron, a phenyl-urea cytokinin, inhibits ergosterol synthesis and attenuates biofilm formation of *Candida albicans*. Res. Sq..

[22] Shamsel-Din H.A., Gizawy M.A., Abdelaziz G. (2020). Molecular docking and preliminary bioevaluation of 99 m Tc-Thiadiazuron as a novel potential agent for cervical cancer imaging. J. Radioanal. Nucl. Chem..

[23] EFSA European Food Safety Authority (2020). Update of the *Xylella* spp. host plant database—Systematic literature search up to 30 June 2019. EFSA J..

[24] Iacopetta D., Ceramella J., Catalano A., Saturnino C., Pellegrino M., Mariconda A., Longo P., Sinicropi M.S., Aquaro S. (2022). COVID-19 at a glance: An up-to-date overview on variants, drug design and therapies. Viruses.

[25] Schneider K., Van der Werf W., Cendoya M., Mourits M., Navas-Cortés J.A., Vicent A., Lansink A.O. (2020). Impact of *Xylella fastidiosa* subspecies *pauca* in European olives. Proc. Natl. Acad. Sci. USA.

[26] EPPO (2019). PM7/24 (4) *Xylella fastidiosa*. EPPO Bull..

[27] Saponari M., Boscia D., Nigro F., Martelli G.P. (2013). Identification of DNA sequences related to *Xylella fastidiosa* in oleander, almond and olive trees exhibiting leaf scorch symptoms in Apulia (Southern Italy). J. Plant Pathol..

[28] Hopkins D.L., Purcell A.H. (2002). *Xylella fastidiosa*: Cause of Pierce’s disease of grapevine and other emergent diseases. Plant Dis..

[29] Chang C.J., Garnier M., Zreik L., Rossetti V., Bové J.M. (1993). Culture and serological detection of the xylem-limited bacterium causing citrus variegated chlorosis and its identification as a strain of *Xylella fastidiosa*. Curr. Microbiol..

[30] Mircetich S.M., Lowe S.K., Moller W.J., Nyland G. (1976). Etiology of almond leaf scorch disease and transmission of the causal agent. Phytopathology.

[31] Mizell R.F., Andersen P.C., Tipping C., Brodbeck B.V. (2020). *Xylella fastidiosa* Diseases and Their Leafhopper Vectors. http://edis.ifas.ufl.edu/pdffiles/IN/IN17400.pdf.

[32] Gould A.B., Lashomb J.H. (2005). Bacterial leaf scorch of shade trees. APSnet Feature.

[33] Cornara D., Morente M., Markheiser A., Bodino N., Tsai C.-W., Fereres A., Redak R.A., Perring T.M., Lopes J.R.S. (2019). An overview on the worldwide vectors of *Xylella fastidiosa*. Entomol. Gen..

[34] Delbianco A., Gibin D., Pasinato L., Morelli M. (2021). Scientific report on the update of the *Xylella* spp. host plant database—Systematic literature search up to 31 December 2020. EFSA J..

[35] Delbianco A., Gibin D., Pasinato L., Morelli M. (2022). Update of the *Xylella* spp. host plant database—Systematic literature search up to 30 June 2021. EFSA J..

[36] Saponari M., Giampetruzzi A., Loconsole G., Boscia D., Saldarelli P. (2019). *Xylella fastidiosa* in olive in Apulia: Where we stand. Phytopathology.

[37] Saponari M., Loconsole G., Cornara D., Yokomi R.K., De Stradis A., Boscia D., Porcelli F. (2014). Infectivity and transmission of *Xylella fastidiosa* by *Philaenus spumarius* (Hemiptera: Aphrophoridae) in Apulia, Italy. J. Econ. Entomol..

[38] Landa B.B., Saponari M., Feitosa-Junior O.R., Giampetruzzi A., Vieira F.J., Mor E., Robatzek S. (2022). *Xylella fastidiosa*’s relationships: The bacterium, the host plants, and the plant microbiome. New Phytol..

[39] Aniţa S., Capasso V., Scacchi S. (2021). Controlling the spatial spread of a *Xylella* Epidemic. Bull. Math. Biol..

[40] Girelli C.R., Hussain M., Verweire D., Oehl M.C., Massana-Codina J., Avendaño M.S., Migoni D., Scortichini M., Fanizzi F.P. (2022). Agro-active endo-therapy treated *Xylella fastidiosa* subsp. *pauca*-infected olive trees assessed by the first ^1^H-NMR-based metabolomic study. Sci. Rep..

[41] Asteggiano A., Franceschi P., Zorzi M., Aigotti R., Dal Bello F., Baldassarre F., Lops F., Carlucci A., Medana C., Ciccarella G. (2021). HPLC-HRMS global metabolomics approach for the diagnosis of “Olive Quick Decline Syndrome” markers in olive trees leaves. Metabolites.

[42] Di Masi S., De Benedetto G.E., Malitesta C., Saponari M., Citti C., Cannazza G., Ciccarella G. (2022). HPLC-MS/MS method applied to an untargeted metabolomics approach for the diagnosis of “olive quick decline syndrome”. Anal. Bioanal. Chem..

[43] Avosani S., Tattoni C., Mazzoni V., Ciolli M. (2022). Occupancy and detection of agricultural threats: The case of *Philaenus spumarius*, European vector of *Xylella fastidiosa*. Agric. Ecosyst. Environ..

[44] Sicard A., Saponari M., Vanhove M., Castillo A.I., Giampetruzzi A., Loconsole G., Saldarelli P., Boscia D., Neema C., Almeida R.P.P. (2021). Introduction and adaptation of an emerging pathogen to olive trees in Italy. Microb. Genom..

[45] Lombardo L., Rizzo P., Novellis C., Vizzarri V. (2021). Preliminary molecular survey of the possible presence of *Xylella fastidiosa* in the upper ionian coasts of Calabria, Italy, through the capture and analysis of its main vector insects. Insects.

[46] Murthy B.N.S., Murch S.J., Saxena P.K. (1998). Thidiazuron: A potent regulator of in vitro plant morphogenesis. Vitr. Cell. Dev. Biol. Plant..

[47] Catalano A., Iacopetta D., Rosato A., Salvagno L., Ceramella J., Longo F., Sinicropi M.S., Franchini C. (2021). Searching for small molecules as antibacterials: Non-cytotoxic diarylureas analogues of triclocarban. Antibiotics.

[48] Catalano A., Rosato A., Salvagno L., Iacopetta D., Ceramella J., Fracchiolla G., Sinicropi M.S., Franchini C. (2021). Benzothiazole-containing analogues of triclocarban with potent antibacterial activity. Antibiotics.

[49] Mulanda E.S., Adero M.O., Amugune N.O., Akunda E., Kinyamario J.I. (2012). High-frequency regeneration of the drought-tolerant tree melia volkensii gurke using low-cost agrochemical thidiazuron. Biotechnol. Res. Int..

[50] Huetteman C.A., Preece J.E. (1993). Thidiazuron: A potent cytokinin for woody plant tissue culture. Plant Cell Tissue Organ Cult..

[51] Yip W.K., Yang S.F. (1986). Effect of thidiazuron, a cytokinin-active urea derivative, in cytokinin-dependent ethylene production systems. Plant Physiol..

[52] Susan J., Murch S.K., Saxena P.K. (1997). Thidiazuron-induced morphogenesis of regal geranium (*Pelargonium domesticum*): A potential stress response. Physiol. Plant..

[53] Akasaka Y., Daimon H., Mii M. (2000). Improved plant regeneration from cultured leaf segments in peanut (*Arachis hypogaea* L.) by limited exposure to thidiazuron. Plant Sci..

[54] Ali H., Khan M.A., Kayani W.K., Khan T., Khan R.S. (2018). Thidiazuron regulated growth, secondary metabolism and essential oil profiles in shoot cultures of Ajuga bracteosa. Ind. Crop. Prod..

[55] Khan I., Khan M.A., Shehzad M.A., Ali A., Mohammad S., Ali H., Alyemeni M.M., Ahmad P. (2020). Micropropagation and production of health promoting lignans in *Linum usitatissimum*. Plants.

[56] Ahmad N., Faisal M., Ahmad A., Alatar A.A., Qahtan A.A., Alok A. (2022). Thidiazuron induced in vitro clonal propagation of *Lagerstroemia speciosa* (L.) Pers.—An important avenue tree. Horticulturae.

[57] Gharari Z., Bagheri K., Sharafi A., Danafar H. (2019). Thidiazuron induced efficient in vitro organogenesis and regeneration of *Scutellaria bornmuelleri*: An important medicinal plant. Vitr. Cell. Develop. Biol. Plant.

[58] Hussain S.A., Ahmad N., Anis M., Hakeem K.R. (2019). Development of an efficient micropropagation system for *Tecoma stans* (L.) Juss. ex Kunth using thidiazuron and effects on phytochemical constitution. Vitr. Cell. Dev. Biol. Plant.

[59] Çelikel F.G., Zhang Q., Zhang Y., Reid M.S., Jiang C.Z. (2021). A cytokinin analog thidiazuron suppresses shoot growth in potted rose plants via the gibberellic acid pathway. Front. Plant Sci..

[60] Unal B.T. (2018). Thidiazuron as an elicitor in the production of secondary metabolite. Thidiazuron: From Urea Derivative to Plant Growth Regulator.

[61] Kumari A., Baskaran P., Van Staden J. (2016). In vitro propagation and antibacterial activity in *Cotyledon Orbiculata*: A valuable medicinal plant. Plant Cell Tissue Organ Cult..

[62] Baskaran P., Kumari A., Van Staden J. (2018). Analysis of the effect of plant growth regulators and organic elicitors on antibacterial activity of *Eucomis autumnalis* and *Drimia robusta* ex vitro-grown biomass. Plant Growth Regul..

[63] Baskaran P., Moyo M., Van Staden J. (2014). In vitro plant regeneration, phenolic compound production and pharmacological activities of *Coleonema pulchellum*. S. Afr. J. Bot..

[64] Pozzi C., Ferrari S., Cortesi D., Luciani R., Stroud R.M., Catalano A., Costi M.P., Mangani S. (2012). The structure of *Enterococcus faecalis* thymidylate synthase provides clues about folate bacterial metabolism. Acta Cryst. D.

[65] Erland L.A., Giebelhaus R.T., Victor J.M., Murch S.J., Saxena P.K. (2020). The morphoregulatory role of thidiazuron: Metabolomics-guided hypothesis generation for mechanisms of activity. Biomolecules.

[66] Wells J.M., Raju B.C., Hung H.-Y., Weisburg W.G., Mandelco-Paul L., Brenner D.J. (1987). *Xylella fastidiosa* gen. nov., sp. nov: Gram-negative, xylem-limited, fastidious plant bacteria related to *Xanthomonas* spp. Int. J. Syst. Evol. Microbiol..

[67] Anbumani S., da Silva A.M., Carvalho I.G., Fischer E.R., de Souza e Silva M., von Zuben A.A.G., Cotta M.A., Carvalhom H.F., de Souza A.A., Janissen R. (2021). Controlled spatial organization of bacterial growth reveals key role of cell filamentation preceding *Xylella fastidiosa* biofilm formation. NPJ Biofilms Microbiomes.

[68] Pierce N.B. (1892). The California Vine Disease: A Preliminary Report of Investigations.

[69] Tumber K.P., Alston J.M., Fuller K.B. (2014). Pierce’s disease costs California $104 million per year. Calif. Agric..

[70] Davis M.J., Purcell A.H., Thomson S.V. (1978). Pierce’s disease of grapevines: Isolation of the causal bacterium. Science.

[71] Hartung J.S. (1994). Citrus variegated chlorosis bacterium: Axenic culture, pathogenicity, and serological relationships with other strains of *Xylella fastidiosa*. Phytopathology.

[72] Blua M.J., Phillips P.A., Redak R.A. (1999). A new sharpshooter threatens both crops and ornamentals. Calif. Agric..

[73] Sorensen J.T., Gill R.J. (1996). Arange extension of Homalodisca coagulate (Say) (Hemiptera: Clypeorrhyncha: Cicadellidae) to Southern California. Pan-Pac. Entomol..

[74] Nigro F., Boscia D., Antelmi I., Ippolito A. (2013). Fungal species associated with a severe decline of olive in southern Italy. J. Plant Pathol..

[75] Panel Plant Health EFSA (2015). Scientific opinion on the risks to plant health posed by *Xylella fastidiosa* in the EU territory, with the identification and evaluation of risk reduction options. EFSA J..

[76] Strona G., Carstens C.J., Beck P.S. (2017). Network analysis reveals why *Xylella fastidiosa* will persist in Europe. Sci. Rep..

[77] Loconsole G., Zicca S., Manco L., El Hatib O., Altamura G., Potere O., Elicio V., Valentini F., Boscia D., Saponari M. (2021). Diagnostic procedures to detect *Xylella fastidiosa* in nursery stocks and consignments of plants for planting. Agriculture.

[78] Purcell A. (2013). Paradigms: Examples from the bacterium *Xylella fastidiosa*. Annu. Rev. Phytopathol..

[79] Morelli M., García-Madero J.M., Jos Á., Saldarelli P., Dongiovanni C., Kovacova M., Saponari M., Baños Arjona A., Hackl E., Webb S. (2021). *Xylella fastidiosa* in olive: A review of control attempts and current management. Microorganisms.

[80] Schaad N.W., Postnikova E., Lacy G., Fatmi M., Chang C.J. (2004). *Xylella fastidiosa* subspecies: X. *fastidiosa* subsp. [correction] *fastidiosa* [correction] subsp. nov., *X. fastidiosa* subsp. *multiplex* subsp. nov., and *X. fastidiosa* subsp. *pauca* subsp. nov. Syst. Appl. Microbiol..

[81] Jolley K.A., Bray J.E., Maiden M.C.J. (2018). Open-access bacterial population genomics: BIGSdb software, the PubMLST.org website and their applications. Wellcome Open Res..

[82] Landa B.B., Castillo A.I., Giampetruzzi A., Kahn A., Román-Écija M., Velasco-Amo M.P., Navas-Cortés J.A., Marco-Noales E., Barbé S., Moralejo E. (2020). Emergence of a plant pathogen in europe associated with multiple intercontinental introductions. Appl. Environ. Microbiol..

[83] Cunty A., Legendre B., de Jerphanion P., Dousset C., Forveille A., Paillard S., Olivier V. (2022). Update of the *Xylella fastidiosa* outbreak in France: Two new variants detected and a new region affected. Eur. J. Plant Pathol..

[84] El Handi K., Hafidi M., Sabri M., Frem M., El Moujabber M., Habbadi K., Haddad N., Benbouazza A., Kubaa R.A., Achbani E.H. (2022). Continuous pest surveillance and monitoring constitute a tool for sustainable agriculture: Case of *Xylella fastidiosa* in Morocco. Sustainability.

[85] Krugner R., Sisterson M.S., Backus E.A., Burbank L.P., Redak R.A. (2019). Sharpshooters: A review of what moves *Xylella fastidiosa*. Austral Entomol..

[86] Santoiemma G., Tamburini G., Sanna F., Mori N., Marini L. (2019). Landscape composition predicts the distribution of *Philaenus spumarius*, vector of *Xylella fastidiosa*, in olive groves. J. Pest Sci..

[87] White S.M., Bullock J.M., Hooftman D.A.P., Chapman D.S. (2017). Modelling the spread and control of *Xylella fastidiosa* in the early stages of invasion in Apulia, Italy. Biol. Invasions.

[88] Cavalieri V., Dongiovanni C., Tauro D., Altamura G., Di Carolo M., Fumarola G., Saponari M., Bosco D. Transmission of the CODIRO strain of *Xylella fastidiosa* by different insect species. Proceedings of the XI European Congress of Press Publications.

[89] Elbeaino T., Yaseen T., Valentini F., Ben Moussa I.E., Mazzoni V., D’onghia A.M. (2014). Identification of three potential insect vectors of *Xylella fastidiosa* in Southern Italy. Phytopathol. Mediterr..

[90] Cavalieri V., Altamura G., Fumarola G., Di Carolo M., Saponari M., Cornara D., Bosco D., Dongiovanni C. (2019). Transmission of *Xylella fastidiosa* subspecies *pauca* sequence type 53 by different insect species. Insects.

[91] Cornara D., Marra M., Tedone B., Cavalieri V., Porcelli F., Fereres A., Purcell A., Saponari M. (2020). No evidence for cicadas’ implication in *Xylella fastidiosa* epidemiology. Èntomol. Gen..

[92] Sevarika M., Rondoni G., Ganassi S., Pistillo O.M., Germinara G.S., De Cristofaro A., Romani R., Conti E. (2022). Behavioural and electrophysiological responses of *Philaenus spumarius* to odours from conspecifics. Sci. Rep..

[93] Rodrigues I., Benhadi-Marín J., Rodrigues N., Baptista P., Pereira J.A. (2022). Olfactory responses to volatile organic compounds and movement parameters of *Philaenus spumarius* and *Cicadella viridis*. J. Appl. Entomol..

[94] Avosani S., Ciolli M., Verrastro V., Mazzoni V. Application of vibrational signals to study and manipulate an insect vector: The case of *Philaenus spumarius* (Hemiptera: Aphrophoridae). Pest Manag. Sci..

[95] Godefroid M., Morente M., Schartel T., Cornara D., Purcell A., Gallego D., Moreno A., Pereira J.A., Fereres A. (2022). Climate Tolerances of *Philaenus spumarius* should be considered in risk assessment of disease outbreaks related to *Xylella fastidiosa*. J. Pest Sci..

[96] Girelli C.R., Angile F., Del Coco L., Migoni D., Zampella L., Marcelletti S., Cristella N., Marangi P., Scortichini M., Fanizzi F.P. (2019). 1H-NMR metabolite fingerprinting analysis reveals a disease biomarker and a field treatment response in *Xylella fastidiosa* subsp. *pauca*-Infected Olive Trees. Plants.

[97] Boscia D., Altamura G., Ciniero A., Di Carolo M., Dongiovanni C., Fumarola G., Giampetruzzi A., Greco P., Notte P., Loconsole G. (2017). Resistenza a *Xylella fastidiosa* in diverse cultivar di olivo. Inf. Agrar..

[98] Baù A., Delbianco A., Stancanelli G., Tramontini S. (2017). Susceptibility of Olea europaea L. varieties to *Xylella fastidiosa* subsp. *pauca* ST53: Systematic literature search up to 24 March 2017. EFSA J..

[99] Camposeo S., Vivaldi G.A., Montemurro C., Fanelli V., Cunill Canal M. (2021). Lecciana, a new low-vigour olive cultivar suitable for super high density orchards and for nutraceutical EVOO production. Agronomy.

[100] El Handi K., Hafidi M., Habbadi K., El Moujabber M., Ouzine M., Benbouazza A., Achbani E.H. (2021). Assessment of ionomic, phenolic and flavonoid compounds for a sustainable management of *Xylella fastidiosa* in Morocco. Sustainability.

[101] Del Coco L., Migoni D., Girelli C.R., Angilè F., Scortichini M., Fanizzi F.P. (2020). Soil and leaf ionome heterogeneity in *Xylella fastidiosa* subsp. *pauca*-infected, non-infected and treated olive groves in Apulia, Italy. Plants.

[102] Pavan S., Vergine M., Nicolì F., Sabella E., Aprile A., Negro C., Fanelli V., Savoia M.A., Montilon V., Susca L. (2021). Screening of olive biodiversity defines genotypes potentially resistant to *Xylella fastidiosa*. Front. Plant Sci..

[103] Bragard C., Dehnen-Schmutz K., Di Serio F., Gonthier P., Jacques M.A., Miret J.A.J., Fejer Justesen A., MacLeod A., Magnusson C.S., Milonas P. (2019). Effectiveness of in planta control measures for *Xylella fastidiosa*. EFSA J..

[104] Scortichini M., Jianchi C., De Caroli M., Dalessandro G., Pucci N., Modesti V., L’Aurora A., Petriccione M., Zampella L., Mastrobuoni F. (2018). A zinc, copper and citric acid biocomplex shows promise for control of *Xylella fastidiosa* subsp. *pauca* in olive trees in Apulia region (Southern Italy). Phytopathol. Mediterr..

[105] Tatulli G., Modesti V., Pucci N., Scala V., L’Aurora A., Lucchesi S., Salustri M., Scortichini M., Loreti S. (2021). Further in vitro assessment and mid-term evaluation of control strategy of *Xylella fastidiosa* subsp. *pauca* in olive groves of Salento (Apulia, Italy). Pathogens.

[106] Cruz L.F., Cobine P.A., De La Fuente L. (2012). Calcium increases *Xylella fastidiosa* surface attachment, biofilm formation, and twitching motility. Appl. Environ. Microbiol..

[107] Cobine P.A., Cruz L.F., Navarrete F., Duncan D., Tygart M., De La Fuente L. (2013). *Xylella fastidiosa* differentially accumulates mineral elements in biofilm and planktonic cells. PLoS ONE.

[108] Navarrete F., De La Fuente L. (2015). Zinc detoxification is required for full virulence and modification of the host leaf ionomer by *Xylella fastidiosa*. Mol. Plant-Microbe Interact..

[109] Dongiovanni C., Fumarola G., Zicca S., Surano A., Di Carolo M., Datome G. In vitro and in vivo effects of ammonium chloride on *Xylella fastidiosa* subsp. pauca infecting olives. Proceedings of the 3rd European Conference on Xylella fastidiosa and XFACTORS Final Meeting.

[110] Hafez M.M., Aboulwafa M.M., Yassien M.A., Hassouna N.A. (2009). Activity of some mucolytics against bacterial adherence to mammalian cells. Appl. Biochem. Biotechnol..

[111] Muranaka L.S., Giorgiano T.E., Takita M.A., Forim M.R., Silva L.F., Coletta-Filho H.D., Machado M.A., de Souza A.A. (2013). *N*-Acetylcysteine in agriculture, a novel use for an old molecule: Focus on controlling the plant–pathogen *Xylella fastidiosa*. PLoS ONE.

[112] Alves de Souza A., Coletta-Filho H.D., Dongiovanni C., Saponari M. N-acetyl-cysteine for controlling Xylella fastidiosa in citrus and olive: Understanding the differences to improve management. Proceedings of the 2nd European Conference on Xylella fastidiosa: How Research Can Support Solutions.

[113] Cattò C., De Vincenti L., Cappitelli F., Datome G., Saponari M., Villa F., Forlani F. (2019). Non-Lethal Effects of N-Acetylcysteine on *Xylella fastidiosa* strain De Donno biofilm formation and detachment. Microorganisms.

[114] Baldassarre F., De Stradis A., Altamura G., Vergaro V., Citti C., Cannazza G., Capodilupo A.L., Dini L., Ciccarella G. (2020). Application of calcium carbonate nanocarriers for controlled release of phytodrugs against *Xylella fastidiosa* pathogen. Pure Appl. Chem..

[115] Bruno G.L., Cariddi C., Botrugno L. (2020). Exploring a sustainable solution to control *Xylella fastidiosa* subsp. *pauca* on olive in the Salento Peninsula, Southern Italy. Crop Prot..

[116] Baró A., Badosa E., Montesinos L., Feliu L., Planas M., Montesinos E., Bonaterra A. (2020). Screening and identification of BP100 peptide conjugates active against *Xylella fastidiosa* using a viability-qPCR method. BMC Microbiol..

[117] Fogaça A.C., Zaini P.A., Wulff N.A., Da Silva P.I., Fázio M.A., Miranda A., Daffre S., Da Silva A.M. (2010). Effects of the antimicrobial peptide gomesin on the global gene expression profile, virulence and biofilm formation of *Xylella fastidiosa*. FEMS Microbiol. Lett..

[118] Arora A.K., Pesko K.N., Quintero-Hernández V., Possani L.D., Miller T.A., Durvasula R.V. (2018). A paratransgenic strategy to block transmission of *Xylella fastidiosa* from the glassy-winged sharpshooter *Homalodisca vitripennis*. BMC Biotechnol..

[119] Maddox C.E., Laur L.M., Tian L. (2010). Antibacterial activity of phenolic compounds against the phytopathogen *Xylella fastidiosa*. Curr. Microbiol..

[120] Bleve G., Gallo A., Altomare C., Vurro M., Maiorano G., Cardinali A., D’Antuono I., Marchi G., Mita G. (2018). In vitro activity of antimicrobial compounds against *Xylella fastidiosa*, the causal agent of the olive quick decline syndrome in Apulia (Italy). FEMS Microbiol. Lett..

[121] Lee S.A., Wallis C.M., Rogers E.E., Burbank L.P. (2020). Grapevine phenolic compounds influence cell surface adhesion of *Xylella fastidiosa* and bind to lipopolysaccharide. PLoS ONE.

[122] Ambrico P.F., Zicca S., Ambrico M., Rotondo P.R., De Stradis A., Dilecce G., Saponari M., Boscia D., Saldarelli P. (2022). Low temperature plasma strategies for *Xylella fastidiosa* inactivation. Appl. Sci..

[123] Catalano A., Iacopetta D., Ceramella J., Scumaci D., Giuzio F., Saturnino C., Aquaro S., Rosano C., Sinicropi M.S. (2022). Multidrug resistance (MDR): A widespread phenomenon in pharmacological therapies. Molecules.

